# SOX9 Knockdown-Mediated FOXO3 Downregulation Confers Neuroprotection Against Ischemic Brain Injury

**DOI:** 10.3389/fcell.2020.555175

**Published:** 2021-03-12

**Authors:** Yiming Deng, Gaoting Ma, Feng Gao, Xuan Sun, Lian Liu, Dapeng Mo, Ning Ma, Ligang Song, Xiaochuan Huo, Hongwei He, Zhongrong Miao

**Affiliations:** ^1^Department of Interventional Neuroradiology, Beijing Tiantan Hospital, Capital Medical University, Beijing, China; ^2^China National Clinical Research Center for Neurological Diseases, Beijing, China; ^3^Center of Stroke, Beijing Institute for Brain Disorders, Beijing, China

**Keywords:** ischemic brain injury, SRY-box transcription factor 9, forkhead box O3, transcription of Cbp/p300-interacting transactivator with Glu/Asp-rich carboxy-terminal domain 2, IκB kinase α

## Abstract

**Background:**

Evidence exists uncovering that SRY-box transcription factor 9 (SOX9) plays a role in ischemic brain injury (IBI). Thus, the current study was conducted to elucidate the specific role of SOX9 and the mechanism by which SOX9 influenced IBI.

**Methods:**

The IBI-associated regulatory factors were searched by bioinformatics analysis. The rat model of IBI was generated using middle cerebral artery occlusion (MCAO) treatment. Neuronal cells were exposed to oxygen-glucose deprivation (OGD). The expressions of SOX9, forkhead box O3 (FOXO3), transcription of Cbp/p300-interacting transactivator with Glu/Asp-rich carboxy-terminal domain 2 (CITED2), and IκB kinase α (IKKα) in OGD-treated neuronal cells were characterized using reverse transcription quantitative polymerase chain reaction (RT-qPCR) assay. The interaction among CITED2, IKKα, and FOXO3 was identified by chromatin immunoprecipitation (ChIP) and dual luciferase reporter gene assays. Gain- and loss-of-function experiments were performed to verify the relationship among SOX9, FOXO3, CITED2, and IKKα and to investigate their functional effects on apoptosis and the inflammatory response of OGD-treated neuronal cells as well as neurological deficit and infarct area of the rat brain.

**Results:**

SOX9, FOXO3, CITED2, and IKKα were highly expressed in OGD-treated neuronal cells. Silencing of SOX9 inhibited OGD-induced neuronal apoptosis and inflammatory response and reduced the neurological deficit and infarct area of the brain in the rats, which were caused by MCAO but were reversed by overexpressing FOXO3, CITED2, or IKKα.

**Conclusion:**

Taken together, our study suggested that upregulation of SOX9 promoted IBI though upregulation of the FOXO3/CITED2/IKKα axis, highlighting a basic therapeutic consideration for IBI treatment.

## Introduction

Intracranial lesions, trauma, or surgery-related injuries can activate immune inflammation and neuroendocrine responses so as to lead to ischemic brain injury (IBI) (Jiang et al., 2017). Ischemic injury is associated with high morbidity and mortality in various diseases such as ischemic stroke (Tang and Zhuang, 2019). Brain injury after focal cerebral ischemia is the most prevalent driver of stroke, which is caused by a variety of pathological processes such as inflammation and apoptosis (Du et al., 2010). In addition, local inflammatory responses resulting from IBI are involved in the activation of resident microglia, leukocytes, and monocyte infiltration (Rajan et al., 2019). Excessive physiological and pathological events such as inflammation and neuronal cell death associated with cerebral ischemia are regulated by multiple signaling pathways (Du et al., 2010). Thus, it is essential to analyze the underlying pathogenic mechanisms of IBI in order to provide new treatment strategies for this disease.

SRY-box transcription factor 9 (SOX9) belongs to the SOX family, which is involved in the regulation of cell fate during embryogenesis (Spokony et al., 2002). SOX9 mutations result in abnormal cell growth (Pritchett et al., 2011). SOX9 is widely expressed in astrocytes and neural progenitor cells in the adult brain and is upregulated in many diseases such as multiple strokes and middle cerebral artery occlusion (MCAO) (Sun et al., 2017). Moreover, the crucial role of SOX9 in treatment of neurological diseases as well as injuries has been confirmed (Wei et al., 2014). It has been suggested that conditional SOX9 ablation is neuroprotective against spinal cord injury (Xu et al., 2018). Additionally, SOX9 is able to upregulate the expression of forkhead box O3 (FOXO3) (Hong et al., 2015). FOXO3 is a member of forkhead transcription factors which are expressed in the brain in different spatial patterns and function as multifunctional regulators of cell proliferation, metabolism, and survival (Accili and Arden, 2004; Fukunaga and Shioda, 2009). A large number of researches have been done showing that FOXO3 participates in the development of IBI (Song et al., 2015; She et al., 2018; Yu et al., 2019). Moreover, FOXO3 is capable of enhancing the transcription of Cbp/p300-interacting transactivator with Glu/Asp-rich carboxy-terminal domain 2 (CITED2) (Bakker et al., 2007). CITED2, also known as MRG1, is a member of the CITED family (Yokota et al., 2003). Evidence has revealed that CITED2 contributes to stroke injury (Huang et al., 2019). Furthermore, CITED2 binds to the promoter of IκB kinase α (IKKα) to elevate its expression (Jayaraman et al., 2016). IKKα, belonging to one of the members of IKK, is able to activate nuclear factor κB (NF-κB) (O’Neill, 2011). More importantly, inhibition of IKKα has been demonstrated to exert a neuroprotective effect on IBI (Wang et al., 2019).

Based on those findings, we proposed that SOX9 may play a crucial role in the progression of IBI through the FOXO3/CITED2/IKKα axis. Both *in vitro* oxygen-glucose deprivation (OGD) and *in vivo* MCAO models were developed to testify to this hypothesis. This study elucidated the functional role of SOX9, which may provide a potential approach for the treatment of IBI.

## Materials and Methods

### Ethics Statement

The experiments involving animals were performed under approval of the Institutional Animal Care and Use Committee of Beijing Tiantan Hospital and in compliance with the recommendations in the Guide for the Care and Use of Laboratory Animals of the National Institutes of Health.

### Microarray-Based Gene Expression Analysis

IBI-related genes were selected through the GeneCards database^1^. KEGG pathway analyses for related genes were performed using KOBAS 3.0^2^. The STRING website^3^ was used to predict the interaction network of genes. The interaction network was imported into the software Cytoscape 3.5.1 for visualization, and the core genes were selected for further research. The relevant transcription factors of genes were predicted through the RNAInter website^4^. The jvenn tool^5^ was applied to obtain the intersection of the transcription factors with genes related to IBI in GeneCards. The core transcriptional regulatory factors of the genes were obtained by analyzing the gene association and interaction through the STRING website. In order to predict the upstream regulatory genes of transcription factors, the GeneCards database and StarBase website^6^ were adopted to find the interaction regulatory genes of transcription factors and select the genes for intersections. Next, gene ontology (GO) functional enrichment analysis of genes was performed through the Panther website^7^. Enriched genes were chosen to predict the associated network through the STRING website again. Whether there was a targeted regulatory relationship between transcription factors and genes was identified on the hTFtarget website^8^.

### Establishment of the MCAO Rat Model

The MCAO model was induced using 105 Sprague-Dawley male rats (weighing 250–300 g) raised in an environment exposed to daylight and had free access to feed and water (Kim et al., 2012). Rats were anesthetized by 5% isoflurane in a mixture containing 30% oxygen and 70% nitrous oxide gas. In total, 0.5% isoflurane in the same gas mixture was applied as anesthesia throughout the procedure. The left internal carotid artery of rats was exposed, followed by inserting a nylon suture and advancing through the carotid bifurcation until the origin of the middle cerebral artery (MCA) was blocked. After 1 h of occlusion, sutures on the blood vessels were taken for reperfusion. Occlusion of the MCA was maintained for 1 h using a nylon suture, followed by reperfusion. Rats allocated to the sham group underwent an identical procedure except MCAO. During surgery, the rectum temperature was maintained at 37 ± 0.5°C using a temperature-adjusted heating pad and heating lamp. Rats were stereotactically injected in the brain with lentivirus carrying negative control for short hairpin RNA (shRNA) (sh-NC), shRNA against IKKα (sh-IKKα), NC for overexpression plasmid (oe-NC), shRNA against SOX9 (sh-SOX9), or IKKα overexpression plasmid (oe-IKKα). All lentiviruses were purchased from Shanghai GenePharma (Shanghai, China). The level of edema-adjusted infarct volume (I_*EA*_) was calculated according to the formula I_*EA*_ = I × (L/R), where I refers to the unadjusted infarct volume, L refers to the contralateral hemisphere volume, and R refers to the ipsilateral hemisphere volume (Nouraee et al., 2019).

### Modified Neurological Severity Scores (mNSS)

mNSS was applied to assess neurological deficits 2 days post-MCAO (Kim et al., 2018). The mNSS system was based on the results of four tests including motor, sensory, balance, and reflex tests. All of those tests were graded using a 0–18 scale (normal: 0; maximal deficit: 18). The higher the score, the more severe the neurobehavioral disorder. Motor scores were determined by (1) suspending a rat by its tail and giving each of the following a score of 0 or 1 (total score 0–3): forelimb flexion, hindlimb flexion, head movement more than 10° with respect to the vertical axis within 30 s; and (2) placing a rat on the floor and giving each of the following scores from 0 to 3: walking normally, 0; inability to walk straightly, 1; circling toward the paretic side, 2; falling on the paretic side. (3) Sensory tests consisted of a placing test (score 0–1) and a proprioceptive test (score 0–1). The beam balance test was adopted to evaluate balance, which was scored from 0 to 6: balancing with a steady posture, 0; grasping the side of the beam, 1; hugging the beam with one limb off the beam, 2; holding the beam with two limbs off the beam or rotating around the beam for over 60 s, 3; trying to balance on the beam but dropping within 20–40 s, 4; trying to balance on the beam but dropping within 20 s, 5; and making no attempt to balance or hang onto the beam, 6. Reflex test scores were determined by awarding the score to the following four items (the maximum possible score was 4): pinna reflex, 0–1; corneal reflex, 0–1; startle reflex, 0–1; and seizures, myoclonus or dystonia, 0–1.

### 2,3,5-Triphenyltetrazolium Chloride (TTC) Staining

Rats from each group (*n* = 5) were anesthetized with 5% isoflurane 2 days post-MCAO. The brain was quickly excised and divided into six consecutive sections (±5, ±3, and ±1 mm from the pontine). Sections were stained with 2% TTC (Sigma-Aldrich, St Louis, MO, United States) at 37°C for 15 min and then fixed in 4% formaldehyde. A digital camera (Kodak DC240, Eastman Kodak Co., Rochester, NY, United States) was used to photograph the infarcted area in each brain slice. The infarct volume was calculated based on the following formula: lesion area of each cross section = (area of contralateral hemisphere/area of ipsilateral hemisphere) × area of ipsilateral infarct. The infarct volume was estimated by multiplying the lesion area of all sections by the sum of the thickness of the sections.

### Morris Water Maze (MWM) Test

The spatial learning and memory of rats were assessed using the MWM test before euthanasia from the 7th and 14th days after MCAO. In brief, the water maze included a circular tub, 120 cm in diameter and 60 cm in height, which was filled with opaque water and a round platform, 6 cm in diameter, that was submerged 1 cm under the water surface. The tub was located in an environment enriched with visual cues external to the maze. Prior to the trials, the rats were allowed to acclimate to the testing environment for 20 min. Invisible platform training was performed for six consecutive days, and each session included four trials. For each trial, the rats were released from the tank wall and allowed to search for and stand on the hidden platform within 60 s. If the rats failed to reach the platform within the allotted time, they would be manually guided. A probe test was followed at 24 h after the training. During the test, the platform was removed, and the performance of rats was recorded for 60 s. The latency of reaching the platform, the time spent in each quadrant, and the number of times the animal crossed the platform area were recorded.

### Reverse Transcription Quantitative Polymerase Chain Reaction (RT-qPCR)

Total RNA from tissues and cells was extracted using the TRIzol reagent (Invitrogen, Carlsbad, CA, United States). The absorbance value was measured at 260 and 280 nm to determine RNA concentration. Then, 1 μg of total RNA was reversely transcribed into cDNA according to the instructions of a PrimeScript RT reagent kit with gDNA Eraser (RRO37A, Takara, Japan). Real-Time PCR assay was developed using an ABI 7500 PCR instrument (Thermo Fisher Scientific, Waltham, MA, United States) based on the instructions of the SYBR Premix Ex Taq (Tli RNaseH Plus) kit (RR820A, Takara). The relative expression of target genes was measured by the 2^–ΔΔ*Ct*^ method normalized to glyceraldehyde-3-phosphate dehydrogenase (GAPDH) (Livak and Schmittgen, 2001). The synthesis of the primer shown in Table 1 was conducted by GenePharma.

**TABLE 1:** Primer sequences for RT-qPCR.

Gene	Primer sequence
SOX9	F: 5′-AAAGGAAGGAAGGGAAGAAAGG-3′
	R: 5′-AATATGGCATCTTTCGATTTCTG-3′
FOXO3	F: 5′-CAACCAAGGAAATGCTCCTC-3′
	R: 5′-CTGTGGCCCTTATCCTTGAA-3′
CITED2	F: 5′-CCGCCCAATGTCATAGACACTGATTTC-3′
	R: 5′-ATTTCTTTCAGCCGCGAGGTTAACC-3′
IKKα	F: 5′-GTCAGGAGAAGTTCGGTTTGA-3′
	R: 5′-ATTCCAGTTTCACGCTCATGGAT-3′
GAPDH	F: 5′-AGGTCGGTGTGAACGGATTTG-3′
	R: 5′-GGGGTCGTTGATGGCAACA-3′

*RT-qPCR, reverse transcription quantitative polymerase chain reaction; SOX9, SRY-box transcription factor 9; FOXO3, forkhead box O3; CITED2, transcription of Cbp/p300-interacting transactivator with Glu/Asp-rich carboxy-terminal domain 2; IKKα, IκB kinase α. GAPDH, glyceraldehyde-3-phosphate dehydrogenase; F, forward; R, reverse.*

### Western Blot Analysis

Total protein from cells was extracted using phenylmethylsulfonyl fluoride and protease inhibitors. After pyrolysis at 4°C for 15 min, the proteins were centrifuged at 15,000 r/min for 15 min. Protein concentration was detected using a bicinchoninic acid protein assay kit (23227, Thermo Fisher Scientific). The proteins were separated by 10% sodium dodecyl sulfate (SDS)-polyacrylamide gel electrophoresis. After separation, the protein was transferred to a polyvinylidene fluoride membrane and then blocked with 5% bovine serum albumin for 1 h. The membrane was probed with diluted primary anti-rabbit antibodies to FOXO3 (1:1,000, ab109629), CITED2 (1:1,000, ab184145), IKKα (1:1,000, ab216327), B-cell lymphoma-2 (Bcl-2) (1:1,000, ab196495), Bcl-2-associated protein X (Bax) (1:1,000, ab32503), caspase 3 (1:500, ab13847), cleaved caspase 3 (1:500, ab49822), neuron-specific enolase (NSE) (1:200, ab180943), S100 calcium binding protein B (S-100B) (1:1,000, ab52642), glial fibrillary acidic protein (GFAP) (1:10,000, ab6842), and β-actin (1:10,000, ab8224) overnight at 4°C. On the next day, the membrane was re-probed with a secondary antibody, goat anti-rabbit antibody to immunoglobulin G (IgG; 1:20,000, ab205718) labeled by horseradish peroxidase for 1.5 h at room temperature. The above-mentioned antibodies were purchased from Abcam Inc. (Cambridge, United Kingdom). A developing solution was added for visualization. Grayscale analysis was performed using ImageJ 1.48u software. The relative grayscale ratio of target protein to β-actin was calculated.

### Cell Culture

Primary cortical neurons were obtained from newborn Sprague-Dawley rats. Rat brain tissues were minced and incubated in 0.125% trypsin for 30 min. The reaction was terminated with Dulbecco Modified Eagle Medium (DMEM)/F12 containing fetal bovine serum. The cell suspension was filtered and centrifuged at 3,000 × *g* for 10 min. The pellet was resuspended in DMEM/F12. Cell concentration was adjusted to 1 × 10^6^/ml. Cells were seeded on 96-well plates coated with 10 mg/L poly-L-lysine (Sigma-Aldrich). After 72 h of culture, 5 μg/ml of arabinosylcytosine was added to the medium to prevent the growth of non-neuronal cells. After 24 h, the medium was replaced with a normal medium and renewed every 72 h. Immunofluorescence was used to examine the expression of microtubule-associated protein 2 (MAP2) (A17409, 1:200, rabbit, ABclonal Technology, MA, United States), following identification of neuronal cells and examination of their purity.

### Cell Infection and OGD Treatment

Lentiviral vectors LV5-GFP (overexpressing vector; #25999, Addgene, Cambridge, MA, United States) and pSIH1-H1-copGFP (silencing of gene vector; LV601B-1, System Biosciences, Palo Alto, CA, United States) were utilized to infect primary cortical neurons. All lentiviruses were purchased from GenePharma. Three shRNA sequences were designed respectively for IKKα, FOXO3, SOX9, and CITED2 including sh-IKKα-1, sh-KKα-2, sh-IKKα-3, sh-FOXO3-1, sh-FOXO3-2, sh-FOXO3-3, SOX9-1, sh-SOX9-2, sh-SOX9-3, sh-CITED2-1, sh-CITED2-2, and sh-CITED2-3. Cells were infected with lentiviral vectors carrying oe-NC, oe-FOXO3, oe-IKKα, oe-CITED2, sh-NC, sh-FOXO3, sh-CITED2, or sh-IKKα 72 h before treatment with OGD. To induce the OGD cell model, cortical neurons were exposed to glucose-free medium containing 5.4 mmol/L KCl, 116.4 mmol/L NaCl, 0.8 mmol/L MgSO_4_, 1.8 mmol/L CaCl_2_, 26.2 mmol/L NaHCO_3_, 2.6 mmol/L NaH_2_PO_4_, and 20.1 mmol/L 4-(2-hydroxyethyl)piperazine-1-ethanesulfonic acid (pH = 7.4) and incubated at 37°C for 2 h in 5% CO_2_ and 95% N_2_. Then 5.6 mmol/L glucose-free Earl’s solution was added to terminate the OGD reaction. Cells were cultured in 5% CO_2_ and 95% O_2_ for 12 h. The medium was renewed with standard medium. After infection for 72 h, cells were exposed to OGD/reoxygenation (Yu et al., 2015).

### Chromatin Immunoprecipitation (ChIP) Assay

ChIP was used to quantify the enrichment of CITED2 protein in the promoter region of IKK gene and to detect the enrichment of FOXO3 in the enhancer region of CITED2. ChIP was carried out using the ChIP assay kit (Millipore Corp., Billerica, MA, United States). Briefly, cells were cross-linked with 4% formaldehyde and resuspended in SDS lysis buffer. Cell nuclei were sheared by ultrasonication. Chromatin components were removed with protein A agarose beads. The lysis buffer was then immunoprecipitated with antibodies to CITED2 (ab6002), FOXO3 (ab12162), H3K4me1 (ab176877, 1:500), and H3K27ac (ab203953) or rabbit antibody to IgG (ab171870) as NC. All antibodies were obtained from Abcam (Cambridge, United Kingdom). Cross-links were reversed, following treatment with proteinase K. The immunoprecipitated DNA was amplified and quantified by RT-qPCR assay.

### Dual-Luciferase Reporter Gene Assay

The artificially synthesized IKKα promoter region fragments were cloned into pGL3-reporter (Promega Corp., Madison, WI, United States). After restriction endonuclease digestion, the IKKα gene promoter fragment was inserted into the pGL3-reporter plasmid with T4 DNA ligase. The constructed luciferase reporter plasmids were co-transfected with oe-CITED2 vector or oe-NC vector into human embryonic kidney 293T (HEK-293T) cells. Cells were cultured in an incubator with 5% CO_2_ and saturated humidity at 37°C. DMEM (Sigma-Aldrich) was renewed every 2–3 days. After transfection for 48 h, the cells were lysed. Luciferase activity was measured using Luminometer TD-20/20 (E5311, Promega) based on the instructions of the Dual-Luciferase Reporter Assay System kit (Promega).

### Flow Cytometry

Flow cytometry was performed to examine neuronal cell apoptosis using the Annexin V-fluorescein isothiocyanate (AnV-FITC) and propidium iodide (PI) kit (BD PharMingen, Santiago, CA, United States) (Yu et al., 2015). After OGD/RP treatment, 1 × 10^6^ cells were collected from each sample and seeded into 24-well plates. Cells were incubated with AnV-FITC at room temperature for 15 min, followed by the addition of PI. After 30 min, cells were ready for flow cytometric analysis, and the rate of apoptosis was determined.

### Terminal Deoxynucleotidyl Transferase-Mediated 2′-Deoxyuridine 5′-Triphosphate-Biotin Nick End-Labeling (TUNEL)

Rat brain tissues were fixed in 4% paraformaldehyde overnight and then embedded in paraffin, followed by cutting into 5 μm sections. Five sections were dewaxed, hydrated, and incubated with 50 μl of 20 μg/ml Proteinase K at 37°C for 30 min. Sections were treated with 0.3% hydrogen peroxide in methanol for 30 min in room temperature to eliminate endogenous peroxidase (POD) activity, followed by incubation with TUNEL solution for 1 h in the dark. Sections were then incubated with 50 μl Converter-POD at 37°C for 30 min and developed with 2% diaminobenzidine for 15 min. The sections were observed under a microscope. After the cells showed brownish yellow nuclei, the reaction was terminated by distilled water. The sections were counterstained with hematoxylin, dehydrated with gradient alcohol (50, 70, 90, and 100% ethanol), cleared with xylene, mounted, and observed under an optical microscope. Ten fields were randomly selected from each section. Those with brownish yellow nuclei were apoptotic positive cells, and those with blue nuclei were normal cells. The ratio of brownish yellow cells to blue cells was taken as the apoptotic rate of neuron cells.

### Statistical Analysis

The data were processed using SPSS 21.0 statistical software (IBM Corp., Armonk, NY, United States). Measurement data were expressed as mean ± standard deviation. Unpaired data in compliance with normal distribution and homogeneity between two groups were compared using an unpaired *t*-test. Comparisons among multiple groups were conducted by one-way analysis of variance (ANOVA) with Tukey’s *post hoc* test. Statistical analysis in relation to time-based measurements within each group was realized using repeated-measures ANOVA, followed by Bonferroni’s *post hoc* test for multiple comparisons. A value of *p* < 0.05 indicated significant difference.

## Results

### IKKα Is Upregulated in IBI, and Downregulation of IKKα Inhibits IBI

**FIGURE 1:**
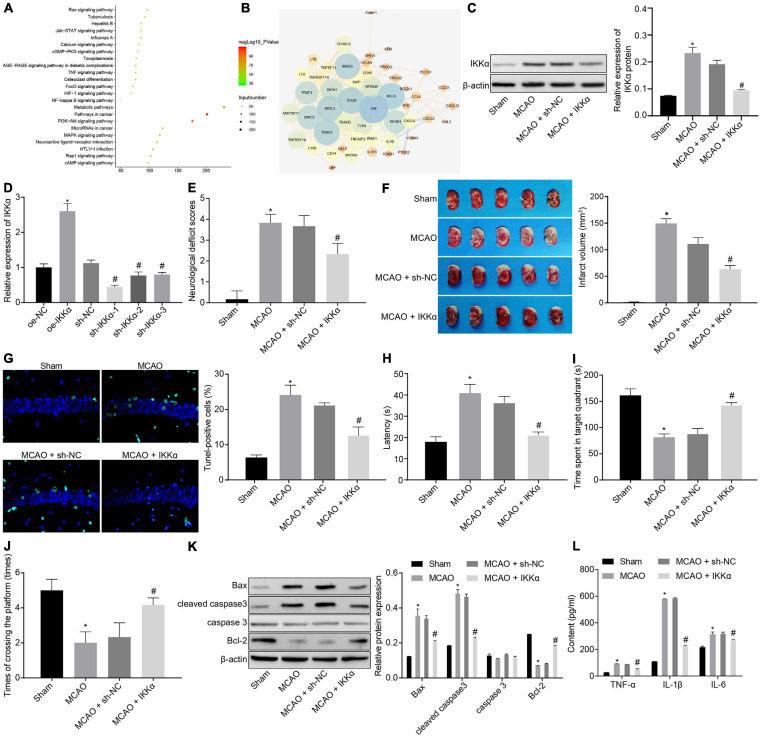
Inhibition of IKKα contributes to prevention of IBI. **(A)** KEGG enrichment analysis on genes on the KOBAS 3.0 website, with the abscissa representing *p* value, the ordinate representing the name of the pathway, and the dot size referring to the number of genes involved in the pathway. **(B)** The network diagram of interaction relations among 50 genes analyzed by the STRING website, with the circle from large to small representing the degree value of the gene, the circle color from blue to orange suggesting the degree from large to small, and the line in the middle referring to the co-expression relationship between genes. **(C)** Western blot analysis for determination of the protein expression of IKKα. **(D)** The overexpressing or silencing effect of lentivirus in neuronal cells determined by RT-qPCR. **(E)** mNSS of rats. **(F)** Results of TTC staining for typical cerebral infarction foci in rats. **(G)** Detection of neuronal cell apoptosis in rats by TUNEL (400×). **(H)** The latency time of rats evaluated by MWM. **(I)** Ratio of time spent by rats staying in the quadrant of the platform. **(J)** The number of times rats passed through the target platform quadrant. **(K)** Expression of apoptosis-related proteins (cleaved caspase 3, Bax, Bcl-2, and caspase 3) normalized to β-actin in rat brain tissues determined by western blot analysis. **(L)** Expression of inflammatory cytokines (TNF-α, IL-1β, and IL-6) in serum of rats measured by ELISA. In panel **(D)**, *p < 0.05 vs. neuronal cells treated with oe-NC; ^#^*p* < 0.05 vs. neuronal cells treated with sh-NC. In panels **(E–L)**, *p < 0.05 vs. sham-operated rats; ^#^*p* < 0.05 vs. MCAO-operated rats treated with sh-NC. Measurement data were expressed as mean ± standard deviation. Unpaired data in compliance with normal distribution and homogeneity between two groups were compared using an unpaired *t*-test. Comparisons among multiple groups were conducted by one-way ANOVA with Tukey’s *post hoc* test. The cell experiment was repeated three times independently.

There were 2,225 genes related to IBI in the GeneCards database, and KOBAS 3.0 was used to conduct KEGG enrichment analysis on the genes (Figure 1A). A previous study has shown that the NF-κB signaling pathway plays a promoting role in the inflammation post-IBI (Banegas et al., 2016). The STRING website was used to predict the network of 50 genes involved in the NF-κB signaling pathway followed by visualization using the software Cytoscape 3.5.1. It was found that tumor necrosis factor (TNF) and IKKα (CHUK) were at the core of the network diagram (Figure 1B). IKKα is a key activating factor in the NF-κB signaling pathway (Tajalli-Nezhad et al., 2019). In order to further enrich the role of IKKα in IBI and its regulatory mechanism, the effect of IKKα on IBI was further explored. First, the MCAO rat model was constructed. Compared with those in the sham-operated rats, the cerebral infarct size and mNSS of the MCAO rats were prominently improved (Figures 1E,F), suggesting that the MCAO rat model was successfully established. Western blot analysis results showed that in comparison to that of sham-operated rats, the expression of IKKα in the brain tissues of the MCAO-exposed rats was markedly increased (Figure 1C). Then lentivirus was used to overexpress or silence the expression of IKKα, and the infection efficiency was detected in neuron cells by RT-qPCR. The results showed that in comparison to cells treated with oe-NC, neuronal cells treated with oe-IKKα revealed a significantly elevated IKKα expression. The expressions of IKKα in neuronal cells treated with sh-IKKα-1, sh-IKKα-2, or sh-IKKα-3 were notably reduced vs. those with sh-NC treatment. In addition, neuronal cells treated with sh-IKKα-1 displayed the lowest expression of IKKα. Therefore, sh-IKKα-1 was selected for follow-up experiments (Figure 1D). Rats were injected with lentivirus carrying sh-IKKα or sh-NC following MCAO treatment. Western blot analysis was applied to determine the IKKα expression. The results revealed that sh-IKKα treatment resulted in a notable reduction in IKKα expression in the MCAO-operated rats (Figure 1C). The mNSS of the MCAO rats was observably higher than that of sham-operated rats. The MCAO-operated rats treated with sh-IKKα had a lower mNSS than those with sh-NC treatment (Figure 1E). TTC staining and TUNEL assay were adopted to detect rat cerebral infarction size and brain tissue apoptosis. The results showed that compared with the sham-operated rats, MCAO-operated rats exhibited an observably increased cerebral infarction area and cell apoptosis. Additionally, MCAO-operated rats treated with sh-IKKα presented a prominent diminished cerebral infarction size and apoptosis in contrast to those with sh-NC treatment (Figures 1F,G). The result of the MWM experiment suggested that the MCAO treatment led to an increased period to reach the platform and to stay in the quadrant where the platform was located and augmented the number of times that rats crossed the platform (Figures 1H–J). At the same time, the expression of apoptosis-related proteins was measured by western blot analysis, which manifested that sh-IKKα treatment resulted in increases in cleaved caspase 3 and Bax protein levels and a decrease in Bcl-2 protein level in the brain tissues of MCAO-operated rats, but there was no significant difference in caspase 3 protein level (Figure 1K). Expression of inflammatory cytokines in serum of rats was examined by enzyme-linked immunosorbent assay (ELISA). It was found that there was an elevated serum expression in TNF-α, interleukin-1β (IL-1β), and IL-6 in MCAO-operated rats in contrast to sham-operated rats. Moreover, sh-IKKα treatment caused reduced serum levels of TNF-α, IL-1β, and IL-6 in the MCAO-operated rats (Figure 1L). These results proved that the expression of IKKα was elevated in IBI and that inhibition of IKKα could inhibit IBI.

### Downregulation of IKKα Inhibits OGD-Induced Neuronal Cell Damage

**FIGURE 2:**
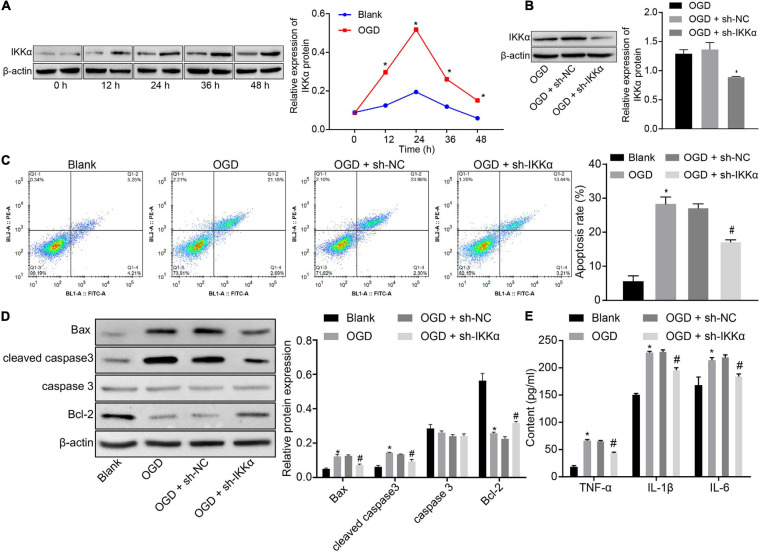
Inhibition of IKKα suppresses OGD-induced neuronal cell apoptosis. **(A)** The protein expression of IKKα normalized to β-actin at different time points after OGD treatment determined by western blot analysis. **(B)** The protein expression of IKKα normalized to β-actin at different time points after knockdown of IKKα measured by western blot analysis. **(C)** The apoptosis of neuronal cells examined by flow cytometry. **(D)** Western blot analysis for the determination of the expression of apoptosis-related proteins (cleaved caspase 3, Bax, Bcl-2, and caspase 3 expression) normalized to β-actin in neuronal cells. **(E)** Expression of inflammatory cytokine (TNF-α, IL-1β, and IL-6) in the cell supernatant determined by ELISA. *p < 0.05 vs. the blank group (neuronal cells without OGD treatment); ^#^*p* < 0.05 vs. OGD-exposed neuronal cells treated with sh-NC. Measurement data were expressed as mean ± standard deviation. Unpaired data in compliance with normal distribution and homogeneity between two groups were compared using an unpaired *t*-test. Comparisons among multiple groups were conducted by one-way ANOVA with Tukey’s *post hoc* test. Data at different time points were compared by repeated-measures ANOVA, followed by Bonferroni’s *post hoc* test. The experiment was repeated three times independently.

In order to study the mechanism of IKKα in IBI, the *in vitro* model of IBI was established by cultures of neuronal cells exposed to OGD. Western blot analysis was adopted to measure the expression of IKKα, which suggested that the expression of IKKα was elevated gradually with the increase of culture time (0–48 h) (Figure 2A). Then lentivirus was used to silence the expression of IKKα. The results of western blot analysis showed that compared with sh-NC-treated neuronal cells under OGD conditions, sh-IKKα-treated neuronal cells under OGD conditions had a markedly reduced IKKα expression (Figure 2B). Apoptosis was detected by flow cytometry. The results revealed notably enhanced neuronal cell apoptosis after exposure to OGD, which was abolished by sh-IKKα (Figure 2C). Apoptosis-related proteins were measured by western blot analysis. The results displayed that OGD treatment led to marked elevations in cleaved caspase 3 and Bax protein levels and a decrease in Bcl-2 protein level, but there was no observable difference in caspase 3 protein level. However, cleaved caspase 3 and Bax protein levels were reduced while Bcl-2 protein level was rescued by sh-IKKα treatment in the OGD-exposed neuronal cells (Figure 2D). ELISA results showed that levels of TNF-α, IL-1β, and IL-6 in the cell supernatant were signally increased in OGD-exposed neuronal cells compared with those in neuronal cells without OGD treatment, but this was reversed by sh-IKKα treatment (Figure 2E). These results indicate that OGD treatment was conducive to an increase of IKKα expression *in vitro*, and inhibition of IKKα suppressed neuronal cell apoptosis induced by OGD treatment.

### CITED2 Aggravates OGD-Induced Neuronal Cell Damage by Upregulating IKKα Expression

**FIGURE 3:**
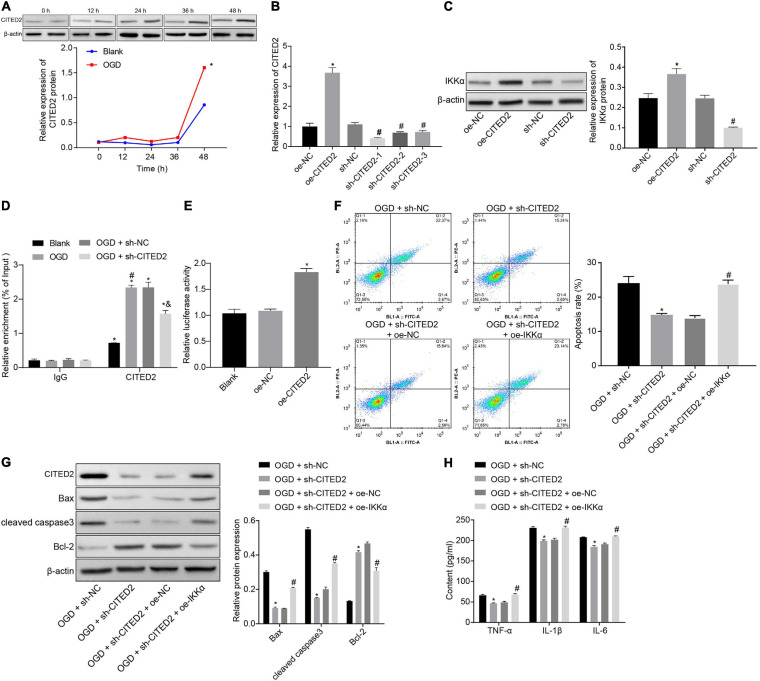
CITED2 upregulates IKKα expression to facilitate OGD-induced neuronal cell apoptosis. **(A)** The protein expression of CITED2 normalized to β-actin at different time points after OGD treatment measured by western blot analysis. **(B)** The mRNA expression of CITED2 in neuronal cells determined by RT-qPCR after overexpression or knockdown of CITED2. **(C)** The protein expression of IKKα normalized to β-actin after overexpression or knockdown of CITED2 measured by western blot analysis. **(D)** The enrichment of CITED2 protein in the promoter region of IKKα detected by ChIP assay in neurons. **(E)** The effect of overexpression of CITED2 on the transcriptional activity of the IKKα promoter region detected by a dual-luciferase reporter gene assay. **(F)** The apoptosis of neurons examined by flow cytometry. **(G)** Western blot analysis for the determination of the expression of apoptosis-related proteins (cleaved caspase 3, Bax, Bcl-2, and caspase 3 expression) normalized to β-actin in neurons. **(H)** Expression of inflammatory cytokine (TNF-α, IL-1β, and IL-6) in cell supernatant determined by ELISA. In panel **(A)**, *p < 0.05 vs. the blank group (neuronal cells without OGD treatment). In panels **(B,C)**, *p < 0.05 vs. neuronal cells treated with oe-NC. In panel **(D)**, *p < 0.05 vs. neuronal cells treated with IgG; ^#^*p* < 0.05 vs. blank group (neuronal cells without OGD treatment); ^&^*p* < 0.05 vs. OGD-exposed neuronal cells treated with sh-NC. In panel **(E)**, *p < 0.05 vs. neuronal cells treated with oe-NC. In panels **(F–H)**, *p < 0.05 vs. OGD-exposed neuronal cells treated with sh-NC; ^#^*p* < 0.05 vs. OGD-exposed neuronal cells treated with sh-CITED2 and oe-NC. Measurement data were expressed as mean ± standard deviation. Unpaired data in compliance with normal distribution and homogeneity between two groups were compared using an unpaired *t*-test. Comparisons among multiple groups were conducted by one-way ANOVA with Tukey’s *post hoc* test. Data at different time points were compared by repeated-measures ANOVA, followed by Bonferroni’s *post hoc* test. The experiment was repeated three times independently.

The upstream regulation mechanism of IKKα was investigated. It has been documented that CITED2 binds to the promoter of IKKα to elevate its expression (Qu et al., 2007). At the same time, CITED2 is highly expressed in IBI and can promote IBI (Huang et al., 2019). Next, the effect of CITED2 on IBI was explored. Western blot analysis was used to determine the expression of CITED2 at different time points during OGD treatment, and it was revealed that the expression of CITED2 was gradually elevated with the increase of treatment time (Figure 3A). Lentivirus was utilized to overexpress or silence the CITED2 expression. RT-qPCR results showed that in comparison to neuronal cells treated with oe-NC, neuronal cells treated with oe-CITED2 showed a markedly increased CITED2 expression. The expression of CITED2 in neuronal cells treated with sh-CITED2-1, sh-CITED2-2, and sh-CITED2-3 was prominently reduced in contrast to those treated with sh-NC treatment. Due to sh-CITED2-1 resulting in the lowest expression of CITED2, this shRNA was selected for follow-up experiments (Figure 3B). The effect of CITED2 on IKKα expression in neuronal cells was measured by western blot analysis. It was demonstrated that expression of IKKα was notably elevated by overexpression of CITED2, but it was reduced by inhibition of CITED2 (Figure 3C), suggesting that CITED2 upregulated IKKα expression. ChIP experiments were performed on neuronal cells to detect the enrichment of CITED2 in the IKKα promoter region, which presented that OGD treatment promoted enrichment of CITED2 in the IKKα promoter region but this was rescued by suppression of CITED2 (Figure 3D). At the same time, a dual-luciferase reporter gene assay was used to verify the transcriptional activity of the IKKα promoter region. Compared with cells treated with oe-NC, cells treated with oe-CITED2 had a notably enhanced luciferase activity (Figure 3E), indicating that CITED2 was able to facilitate the transcription activity of IKKα. Flow cytometry and western blot analysis were employed to examine neuronal cell apoptosis. It was proved that silencing of CITED2 prominently reduced neuronal cell apoptosis under OGD conditions, accompanied by decreased protein levels of cleaved caspase 3 and Bax as well as increased Bcl-2 protein level, but those were abolished by upregulated IKKα (Figures 3F,G). The expression of inflammatory cytokines in the cell supernatant was detected by ELISA. It was validated that the levels of TNF-α, IL-1β, and IL-6 in the cell supernatant were dramatically reduced by inhibition of CITED2 under OGD conditions, whose effect was revoked *via* overexpression of IKKα (Figure 3H). Those results demonstrated that CITED2 promoted OGD-induced neuronal cell apoptosis by upregulating the IKKα expression.

### FOXO3 Accelerates OGD-Induced Neuronal Cell Apoptosis by Increasing CITED2 and IKKα Expressions

**FIGURE 4:**
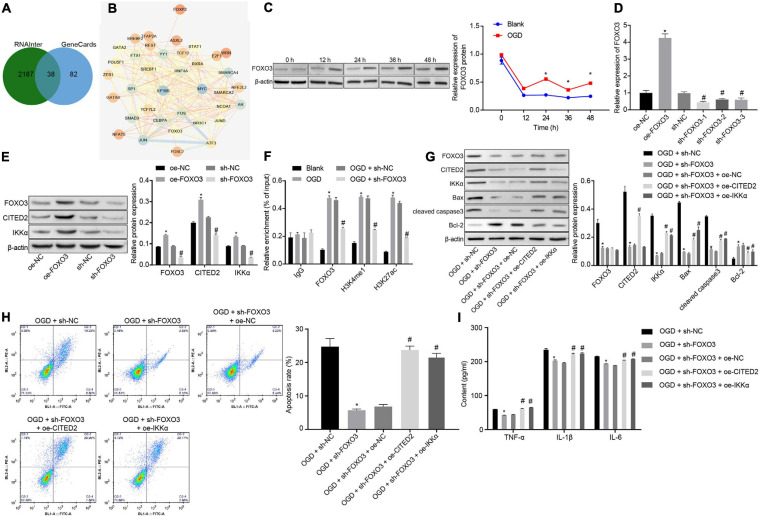
FOXO3 upregulates CITED2 and IKKα expressions to enhance OGD-induced neuronal cell apoptosis. **(A)** Venn plot of the intersection of CITED2-related transcription factors and brain ischemia injury-related genes in GeneCards predicted on the RNAInter website. **(B)** The network diagram of interaction relationships among 38 genes analyzed on the STRING website, with the color of circles from blue to orange indicating degree and the line in the middle of circles referring to the co-expression relationship between genes. **(C)** The protein expression of FOXO3 normalized to β-actin at different time points after OGD treatment determined by western blot analysis. **(D)** The mRNA expression of FOXO3 in neuronal cells determined by RT-qPCR after overexpression or knockdown of FOXO3. **(E)** The protein expressions of FOXO3, CITED2, and IKKα normalized to β-actin after overexpression or knockdown of FOXO3 measured by western blot analysis. **(F)** The enrichment of FOXO3, H3K4me1, and H3K27ac in the enhancer region of CITED2 detected by ChIP assay in neurons. **(G)** Western blot analysis for the determination of the expression of apoptosis-related proteins (cleaved caspase 3, Bax, Bcl-2, and caspase 3 expression) normalized to β-actin in neurons. **(H)** The apoptosis of neurons in each group examined by flow cytometry. **(I)** Expression of inflammatory cytokine (TNF-α, IL-1β, and IL-6) in the cell supernatant determined by ELISA. In panel **(C)**, *p < 0.05 vs. the blank group (neuronal cells without OGD treatment). In panels **(D,E)**, *p < 0.05 vs. neuronal cells treated with oe-NC; ^#^*p* < 0.05 vs. neuronal cells treated with sh-NC. In panel **(F)**, *p < 0.05 vs. neuronal cells treated with IgG; ^#^*p* < 0.05 vs. neuronal cells treated with the blank group (neuronal cells without OGD treatment); In panels **(G**–**I)**, *p < 0.05 vs. OGD-exposed neuronal cells treated with sh-NC; ^#^*p* < 0.05 vs. OGD-exposed neuronal cells treated with sh-FOXO3 and oe-NC. Measurement data were expressed as mean ± standard deviation. Unpaired data in compliance with normal distribution and homogeneity between two groups were compared using an unpaired *t*-test. Comparisons among multiple groups were conducted by one-way ANOVA with Tukey’s *post hoc* test. Data at different time points were compared by repeated-measures ANOVA, followed by Bonferroni’s *post hoc* test. The experiment was repeated three times independently.

In total, 120 CITED2-related transcription factors were obtained from the RNAInter website, and 38 candidate genes were found in the intersection of transcription factors and genes related to IBI in GeneCards (Figure 4A). The interactions of the 38 genes were analyzed through the STRING website. It was found that 16 transcription factors were at the core position of the network map (degree ≥ 15) (Figure 4B). A previous study has shown that FOXO3 binds to the enhancer of CITED2 and promotes enhancer activity so as to elevate its expression (Eijkelenboom et al., 2013). Thus, whether FOXO3 regulates the CITED2/IKKα axis to be involved in the progression of IBI was investigated in the present study. Western blot analysis was applied to determine the expression of FOXO3 at different time points during OGD treatment, and it was shown that the expression of FOXO3 was gradually increased with the increase of treatment time (Figure 4C). Lentivirus was utilized to overexpress or silence FOXO3 expression. RT-qPCR results presented that treatment with oe-FOXO3 resulted in an elevation in FOXO3 expression in neuronal cells while treatment with sh-FOXO3-1, sh-FOXO3-2, or sh-FOXO3-3 caused a decreased expression of FOXO3 in neuronal cells. Among these shRNAs, sh-FOXO3-1 with better silencing efficiency was selected for subsequent experiments (Figure 4D). The effect of FOXO3 on expressions of CITED2 and IKKα was examined by western blot analysis (Figure 4E), which manifested that expressions of CITED2 and IKKα in neuronal cells were significantly increased by overexpression of FOXO3, which was markedly reduced by silencing of FOXO3. It was demonstrated that FOXO3 contributed to the upregulation of CITED2 and IKKα. A ChIP experiment was conducted in neuronal cells to detect the enrichment of FOXO3, H3K4me1, and H3K27ac in the CITED2 enhancer region (Figure 4F). It was validated that the enrichment of FOXO3, H3K4me1, and H3K27ac in the CITED2 enhancer region of neuronal cells with OGD treatment was signally promoted in comparison with those without OGD treatment. In addition, silencing of FOXO3 resulted in diminished enrichment of FOXO3, H3K4me1, and H3K27ac in the CITED2 enhancer region of OGD-exposed neuronal cells. Western blot analysis results suggested that FOXO3 knockdown in OGD-exposed neuronal cells resulted in a prominent reduction in the expression of CITED2 and IKKα. Reduced expression of IKKα was rescued by upregulation of CITED2. Additionally, reduced expression of IKKα by inhibition of FOXO3 was rescued by treatment with oe-IKKα (Figure 4G). It was proved that after OGD treatment, suppression of FOXO3 could downregulate the expressions of CITED2 and IKKα, but overexpression of CITED2 or IKKα had no effect on FOXO3 expression. Flow cytometry and Western blot analysis were adopted to detect neuronal cell apoptosis. It was shown that silencing of FOXO3 prominently inhibited neuronal cell apoptosis under OGD conditions, accompanied by diminished protein levels of cleaved caspase 3 and Bax as well as elevated Bcl-2 protein level, but those effects were revoked by overexpressed CITED2 or IKKα (Figures 4G,H). The expression of inflammatory cytokines in the cell supernatant was examined by ELISA. It was found that the levels of TNF-α, IL-1β, and IL-6 in the cell supernatant were observably reduced by inhibition of FOXO3 under OGD conditions, but the effect caused by suppression of FOXO3 was reversed by overexpression of CITED2 or IKKα (Figure 4I). Those results demonstrated that FOXO3 stimulated OGD-induced neuronal cell apoptosis by upregulating CITED2 and IKKα expressions.

### SOX9 Upregulates the FOXO3/CITED2/IKKα Axis to Promote OGD-Induced Neuronal Cell Apoptosis

**FIGURE 5:**
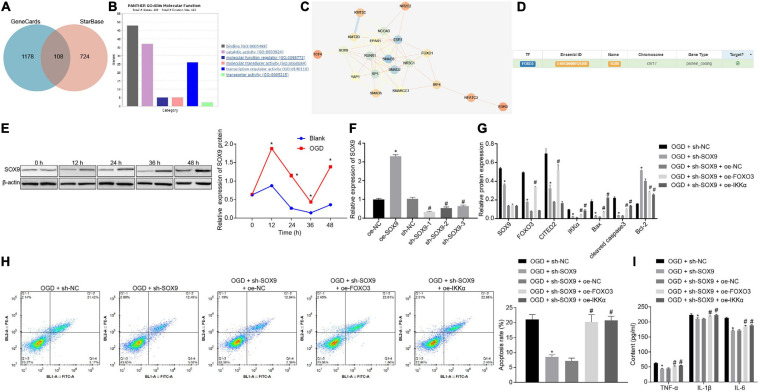
SOX9 enhances OGD-induced neuronal cell apoptosis *via* upregulating the FOXO3/CITED2/IKKα axis. **(A)** Venn plot of the intersection of the regulatory gene of FOXO3 in GeneCards predicted on the RNAInter website. **(B)** GO enrichment analysis of genes through the Panther website. **(C)** The network diagram of interaction relationships among 26 genes analyzed on the STRING website, with the color of circles from blue to orange referring to degree and the line in the middle of circles indicating the co-expression relationship between genes. **(D)** The binding relationship between FOXO3 and SOX9 identified on the hTFtarget website. **(E)** The protein expression of SOX9 normalized to β-actin at different time points after OGD treatment determined by western blot analysis. **(F)** The mRNA expression of SOX9 in neuronal cells determined by RT-qPCR after silencing of SOX9. **(G)** Western blot analysis for measurement of expression of apoptosis-related proteins (cleaved caspase 3, Bax, Bcl-2, and caspase 3 expression) normalized to β-actin in neurons. **(H)** The apoptosis of neurons examined by flow cytometry. **(I)** Expression of inflammatory cytokine (TNF-α, IL-1β, and IL-6) in the cell supernatant assessed by ELISA. In panel **(E)**, *p < 0.05 vs. the blank group (neuronal cells without OGD treatment). In panel **(F)**, *p < 0.05 vs. neuronal cells treated with oe-NC; ^#^*p* < 0.05 vs. neuronal cells treated with sh-NC. In panels **(G**–**I)**, *p < 0.05 vs. OGD-exposed neuronal cells treated with sh-NC; ^#^*p* < 0.05 vs. OGD-exposed neuronal cells treated with sh-SOX9 and oe-NC. Measurement data were expressed as mean ± standard deviation. Comparisons among multiple groups were conducted by one-way ANOVA with Tukey’s *post hoc* test. Data at different time points were compared by repeated-measures ANOVA, followed by the Bonferroni’s *post hoc* test. The experiment was repeated three times independently.

In order to predict the upstream regulatory genes of FOXO3, we searched the interaction regulatory genes of FOXO3 through the GeneCards database and StarBase website and obtained 108 genes from the intersection (Figure 5A). GO enrichment analysis of genes was conducted through the Panther website, and it was found that 26 genes were involved in the molecular functional process of transcriptional regulatory activity of FOXO3 (Figure 5B). By using the STRING website to predict the correlation network of 26 genes, it was revealed that eight genes were at the core of the network (degree ≥ 7) (Figure 5C). Meanwhile, SOX9 has been proven to be able to increase the expression of FOXO3 (Ludbrook et al., 2016). A binding relationship between FOXO3 and SOX9 was confirmed through the hTFtarget website (Figure 5D). Next, whether the role of SOX9 in IBI was related to the FOXO3/CITED2/IKK axis was further investigated. Western blot analysis was adopted to measure the expression of SOX9 at different time points during OGD treatment, and it was shown that the expression of SOX9 was gradually upregulated with the increase of treatment time (Figure 5E). Lentivirus was utilized to silence the SOX9 expression. RT-qPCR results revealed that neuronal cells treated with sh-SOX9-1, sh-SOX9-2, or sh-SOX9-3 presented a decrease in expression of FOXO3, especially those treated with sh-SOX9-1. Thus, sh-SOX9-1 was selected for subsequent experiments (Figure 5F). Infected cells were treated with OGD, and the effect of SOX9 on expressions of FOXO3, CITED2, and IKKα in OGD-exposed neuronal cells was examined by western blot analysis (Figure 5G). The results displayed that silencing of SOX9 led to reduced expressions of FOXO3, CITED2, and IKKα under OGD conditions, but the reduced expression caused by silencing of SOX9 was reversed by overexpressed FOXO3. However, SOX9 expression was not affected by overexpressed FOXO3. In addition, oe-IKKα treatment rescued the reduced expression of IKKα resulting from the inhibition of SOX9, but IKKα overexpression caused no notable difference in the expressions of SOX9, FOXO3, and CITED2. It was suggested that under OGD conditions, downregulating SOX9 could reduce the expression of FOXO3, CITED2, and IKKα, while overexpressing FOXO3 or IKKα had no effect on SOX9 expression. Flow cytometry and western blot analysis results demonstrated that SOX9 silencing markedly restrained neuronal cell apoptosis and neuronal damage under OGD conditions, as evidenced by reduced protein levels of cleaved caspase 3, Bax, and neuronal damage markers (NSE, S-100B, and GFAP) and increased Bcl-2 protein level, which were however neutralized by overexpression of FOXO3 or IKKα (Figures 5G,H and Supplementary Figure 1). The expression of inflammatory cytokines in the cell supernatant was assessed by ELISA, which showed that the levels of TNF-α, IL-1β, and IL-6 in the cell supernatant under OGD conditions were notably decreased by the suppression of SOX9, but those were rescued by upregulated FOXO3 or IKKα (Figure 5I). Those results validated that SOX9 facilitated OGD-induced neuronal cell apoptosis by upregulating the FOXO3/CITED2/IKKα axis.

### SOX9 Aggravates IBI in Rats *via* Upregulating the FOXO3/CITED2/IKKα Axis

**FIGURE 6:**
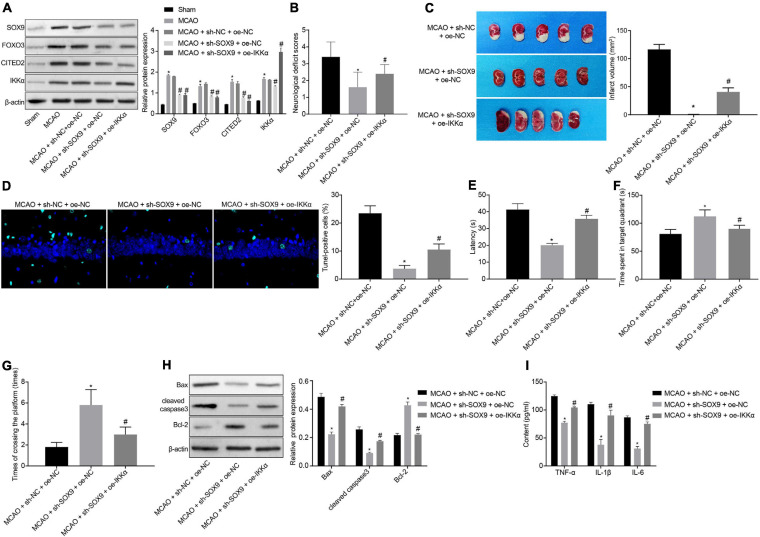
SOX9 facilitates IBI in rats though upregulation of the FOXO3/CITED2/IKK axis. **(A)** Western blot analysis for measurement of expression of SOX9, FOXO3, CITED2, and IKKα normalized to β-actin in brain tissues of rats. **(B)** mNSS of rats. **(C)** Results of TTC staining for typical cerebral infarction foci in rats. **(D)** Examination of neuronal cell apoptosis in rats by TUNEL (400×). **(E)** The latency time of rats assessed by MWM. **(F)** Ratio of time spent by rats staying in the quadrant of the platform. **(G)** The number of times rat passed through the target platform quadrant. **(H)** Expression of apoptosis-related proteins (cleaved caspase 3, Bax, Bcl-2, and caspase 3 expression) normalized to β-actin in rat brain tissues determined by western blot analysis. **(I)** Expression of inflammatory cytokines (TNF-α, IL-1β, and IL-6) in brain tissues of rats measured by ELISA. In panel **(A)**, *p < 0.05 vs. sham-operated rats; ^#^*p* < 0.05 vs. MCAO-operated rats treated with sh-NC; In panels **(B–I)**, *p < 0.05 vs. MCAO-operated rats treated with sh-NC and oe-NC; ^#^*p* < 0.05 vs. MCAO-operated rats treated with sh-SOX9 and oe-NC. Measurement data were expressed as mean ± standard deviation. Comparisons among multiple groups were conducted by one-way ANOVA with Tukey’s *post hoc* test. The experiment was repeated three times independently.

The effect of SOX9 in IBI by regulating the FOXO3/CITED2/IKK axis was further explored *in vivo*. Rats were injected with lentivirus into the brains. The protein levels of SOX9, FOXO3, CITED2, and IKKα in brain tissues of rats were measured by Western blot analysis. It was shown that MCAO treatment was conducive to elevating the protein levels of SOX9, FOXO3, CITED2, IKKα, and neuronal damage markers (NSE, S-100B, and GFAP). Silencing of SOX9 could result in marked decreases in the protein levels of SOX9, FOXO3, CITED2, IKKα, and neuronal damage markers (NSE, S-100B, and GFAP) in the brain tissues of MCAO-operated rats. Overexpressing of IKKα could reverse the decreased protein levels of IKKα and neuronal damage markers (NSE, S-100B, and GFAP) but had no effect on SOX9, FOXO3, and CITED2 protein levels in the brain tissues of MCAO-operated rats (Figure 6A and Supplementary Figure 2). The results of mNSS showed that silencing of SOX9 led to diminished mNSS of MCAO-operated rats, which was abolished by upregulated IKKα (Figure 6B). The cerebral infarction area was observed by TTC staining, and brain tissue apoptosis was examined by TUNEL. The results displayed that SOX9 silencing inhibited cerebral infarction area and cell apoptosis in MCAO-operated rats, but this inhibitory effect was abolished by overexpression of IKKα (Figures 6C,D). The result of the MWM experiment suggested increased time to reach the platform and to stay in the quadrant where the platform was located and a boosted number of times that MCAO-operated rats crossed the platform after silencing of SOX9, both of which were reversed by upregulated IKKα (Figures 6E–G). The levels of apoptosis-related proteins were determined by western blot analysis, which revealed that sh-SOX9 treatment led to notable elevations in cleaved caspase 3 and Bax protein levels and a reduction in Bcl-2 protein level in the brain tissues of MCAO-operated rats, but those effects were abolished by sh-IKKα treatment (Figure 6H). The results of ELISA showed that inhibition of SOX9 markedly restrained the levels of TNF-α, IL-1β, and IL-6 in brain tissues of MCAO-operated rats, but upregulated IKKα reversed the inhibitory effect of SOX9 silencing on inflammatory cytokines (Figure 6I). All these results demonstrated that SOX9 worsened IBI in rats by elevating the FOXO3/CITED2/IKKα axis.

## Discussion

It is known that inflammation and apoptosis are typical pathological mechanisms of IBI, so inhibition of inflammation and neuronal cell apoptosis in the brain may be effective therapeutic interventions for IBI (Chien et al., 2016; El Khashab et al., 2019). We focus on discussion of inflammation and neuronal cell apoptosis in *in vivo* and *in vitro* models of IBI in order to investigate effects of the SOX9/FOXO3/CITED2/IKKα axis on IBI development. Collectively, findings of the study revealed that SOX9 deteriorated IBI by promoting inflammation and neuronal cell apoptosis through upregulation of the FOXO3/CITED2/IKKα axis.

The MCAO and OGD exposure methods were used to successfully establish the model of IBI *in vivo* and *in vitro*. The transcription factor NF-κB functions as a key regulator of hundreds of genes associated with cell survival and inflammation, which is activated during cerebral ischemia and is conductive to neuronal cell death (Schneider et al., 1999; Ridder and Schwaninger, 2009). There is ample evidence that IKKα is a key activator in the NF-κB signaling pathway (El Khashab et al., 2019). The first finding of this study was that IKKα was expressed at a high level in brain tissues of rats treated with MCAO. Further analysis demonstrated that downregulation of IKKα could inhibit IBI in rats and OGD-induced neuronal cell damage, as evidenced by reduced levels of cleaved caspase 3, Bax, TNF-α, IL-1β, and IL-6 and increased Bcl-2 level in brain tissues of rats and neuronal cells. Caspase-3 belongs to the cysteine protease family, which is responsible for most of the proteolysis during apoptosis. Thus, determination of cleaved caspase-3 is thought to be a reliable marker of apoptosis (Crowley and Waterhouse, 2016). Bax is a member of the Bcl-2 family and serves as a core regulator of the intrinsic apoptosis pathway (Pena-Blanco and Garcia-Saez, 2018). Bcl-2 functions as an anti-apoptotic factor to modulate intrinsic apoptosis (Cui and Placzek, 2018). Pro-inflammatory cytokines, such as TNF-α, IL-1β, and IL-6, are produced in the central nervous system and exert great effects on neuroprotection, which have been proven to be elevated in traumatic brain injury (Carlson et al., 1999; Chen et al., 2007). Consistent with our results, Wang et al. (2019) have demonstrated that inhibition of IKKα has contributed to protection against IBI, accompanied by elevated Bcl-2 expression and decreased Bax expression. In addition, recruitment of CITED2 is found in the promoter of IKKα and elevates its expression (Jayaraman et al., 2016). Moreover, increased expression of CITED2 has been presented in rats with transient forebrain ischemia (Sun et al., 2006). The findings of our study implied that silencing of CITED2 could downregulate IKKα to reduce the levels of cleaved caspase 3, Bax, TNF-α, IL-1β, and IL-6 and to increase the level of Bcl-2, thus restraining OGD-induced neuronal cell damage and reducing inflammatory responses. Similar to our result, findings obtained from previous studies have also manifested that overexpression of CITED2 contributes to neuronal cell death while inhibition of CITED2 confers neuroprotection (Gonzalez et al., 2008; Huang et al., 2019). Furthermore, CITED2 is upregulated by FOXO3, which can bind to the enhancer of CITED2 and enhance the enhancer activity (Eijkelenboom et al., 2013). The current study verified that FOXO3 was highly expressed in OGD-induced neuronal cells. Evidence has revealed that activation of FOXO3 precedes delayed apoptosis of neuronal cells in the vulnerable hippocampal regions, demonstrating its crucial role in the progression of IBI (Fukunaga and Shioda, 2009). For example, FOXO3 enhances cell autophagy so as to promote ischemic stroke (Yu et al., 2019). Additionally, overexpressed FOXO3 plays a deteriorative role in ischemic stroke by stimulating neuronal cell death (Guo et al., 2018). Moreover, it has been supported that inhibition of FOXO3a plays a neuroprotective effect in ischemic stroke (She et al., 2018). Additionally, our data also presented that downregulation of FOXO3 could impede neuronal damage and inflammatory response in OGD-induced neuronal cells though inhibition of the CITED2/IKKα axis. Furthermore, SOX9 is confirmed to be able to upregulate FOXO3 expression (Hong et al., 2015). Overexpressed SOX9 had been displayed in rats treated with MCAO. More importantly, a previously conducted study has suggested that upregulation of SOX9 has the potential to worsen hepatic ischemia/reperfusion injury *via* promoting inflammation and apoptosis (Fan et al., 2018). However, silencing of SOX9 contributes to neuroprotection and recovery after stroke (Hong et al., 2015). These results are partially consistent with our study that inhibition of SOX9 resulted in suppressed neuronal damage and inflammatory response in OGD-induced neuronal cells. Together with our experiment, we concluded that SOX9 could deteriorate IBI by upregulating the FOXO3/CITED2/IKKα axis.

## Conclusion

Taken together, the aforementioned findings elucidated the deteriorative effect of SOX9 on IBI and supported that silencing of SOX9 contributed to protection from IBI by suppressing neuronal damage and inflammatory response *via* inhibition of the FOXO3/CITED2/IKKα axis. Thus, targeting SOX9 or the small molecule inhibitor of SOX9 might serve as a promising therapeutic strategy for the treatment of IBI.

## Data Availability Statement

The original contributions presented in the study are included in the article/Supplementary Material, further inquiries can be directed to the corresponding author/s.

## Ethics Statement

The animal study was reviewed and approved by the Institutional Animal Care and Use Committee of Beijing Tiantan Hospital.

## Author Contributions

YD conceived and designed the research. GM performed the experiments. FG and XS interpreted the results of the experiments. LL and DM analyzed the data. NM and LS prepared the figures. XH drafted the manuscript. HH and ZM edited and revised the manuscript. All authors read and approved the final manuscript.

## Conflict of Interest

The authors declare that the research was conducted in the absence of any commercial or financial relationships that could be construed as a potential conflict of interest.

## Footnotes

1 https://www.genecards.org/

2 http://kobas.cbi.pku.edu.cn/

3 https://string-db.org/

4 http://www.rna-society.org/rnainter/

5 http://jvenn.toulouse.inra.fr/app/

6 http://starbase.sysu.edu.cn/

7 http://www.pantherdb.org/

8 http://bioinfo.life.hust.edu.cn/hTFtarget#!/

## Supplementary Material

Supplementary Figure 1 SOX9 silencing restrains neuronal damage through upregulating the FOXO3/IKKα. Western blot analysis of neuronal damage markers (NSE, S-100B, and GFAP) normalized to β-actin in OGD-exposed neurons after infection with lentivirus expressing sh-SOX9, and/or oe-FOXO3 or oe-IKKα.

Supplementary Figure 2 SOX9 silencing restrains neuronal damage through upregulating the FOXO3/IKKα in MCAO-operated rats. Western blot analysis of neuronal damage markers (NSE, S-100B, and GFAP) normalized to β-actin in MCAO-operated rats after delivery of lentivirus expressing sh-SOX9 and/or oe-IKKα.

## Abbreviations

ANOVA: analysis of variance
AnV-FITC: Annexin V–fluorescein isothiocyanate
BCA: bicinchoninic acid
ChIP: chromatin immunoprecipitation
CITED2: carboxy-terminal domain 2
DAB: diaminobenzidine
FBS: fetal bovine serum
FOXO3: forkhead box O3
HEK-293T: human embryonic kidney 293T
HRP: horseradish peroxidase
IBI: ischemic brain injury
IKKα: IκB kinase α
MCAO: middle cerebral artery occlusion
mNSS: modified neurological severity scores
MWM: Morris water maze
OGD: oxygen-glucose deprivation
PMSF: phenylmethylsulfonyl fluoride
POD: peroxidase
PVDF: polyvinylidene fluoride
RT-qPCR: reverse transcription quantitative polymerase chain reaction
SD: Sprague-Dawley
SDS: sodium dodecyl sulfate
SDS-PAGE: sulfate-polyacrylamide gel electrophoresis
SOX9: SRY-box transcription factor 9
TTC: 2,3,5-triphenyltetrazolium chloride
TUNEL: terminal deoxynucleotidyl transferase (TDT)-mediated dUTP-biotin nick end-labeling.
