# Supplementary figures and images for: SOX9 Knockdown-Mediated FOXO3 Downregulation Confers Neuroprotection Against Ischemic Brain Injury
Source: Front Cell Dev Biol. 2021 Mar 12;8:555175. doi: 10.3389/fcell.2020.555175 (PMC8006459; doi:10.3389/fcell.2020.555175)

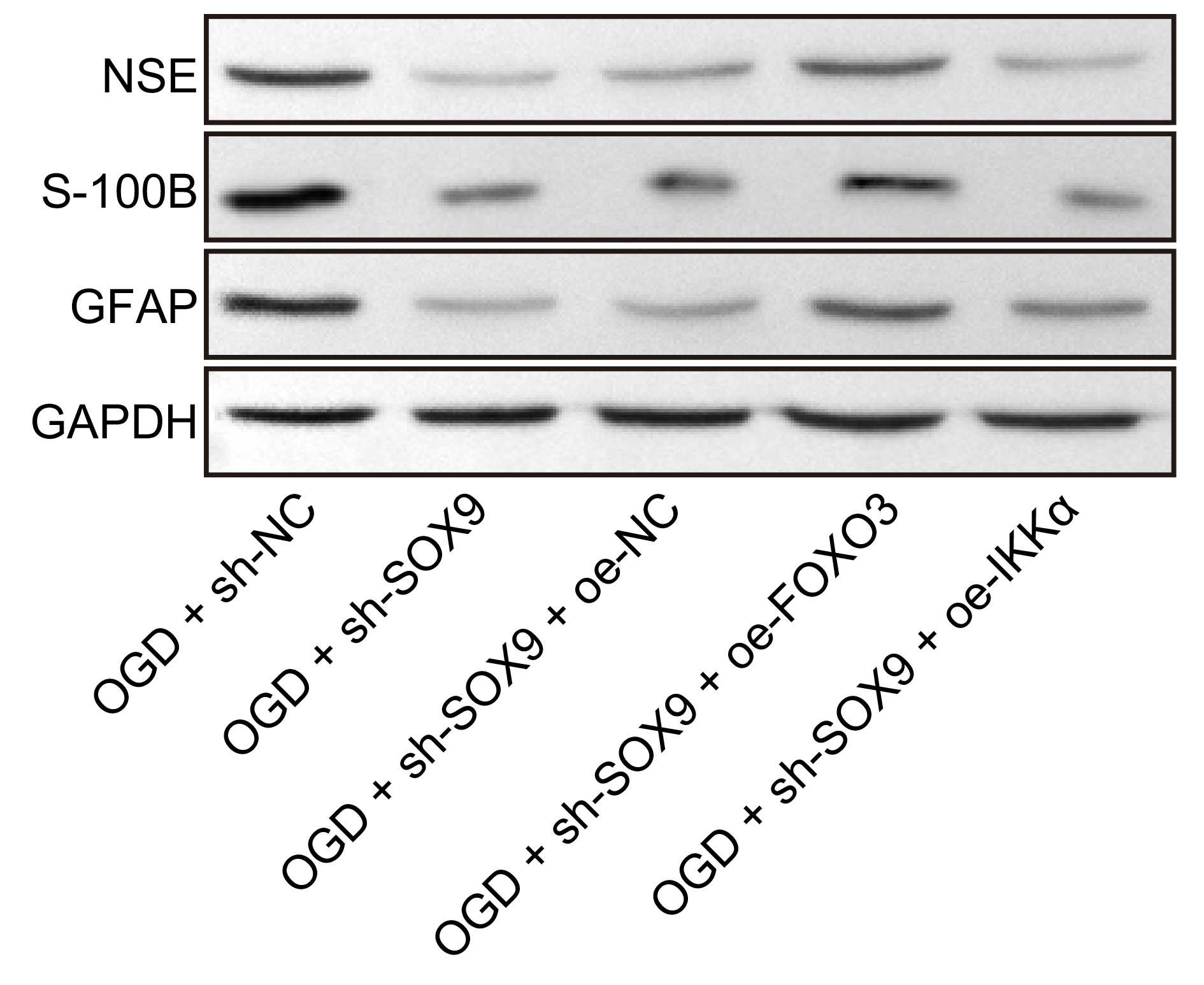

Supplement: Supplementary Figure 1 — SOX9 silencing restrains neuronal damage through upregulating the FOXO3/IKKα. Western blot analysis of neuronal damage markers (NSE, S-100B, and GFAP) normalized to β-actin in OGD-exposed neurons after infection with lentivirus expressing sh-SOX9, and/or oe-FOXO3 or oe-IKKα. [file Image_1.JPEG]

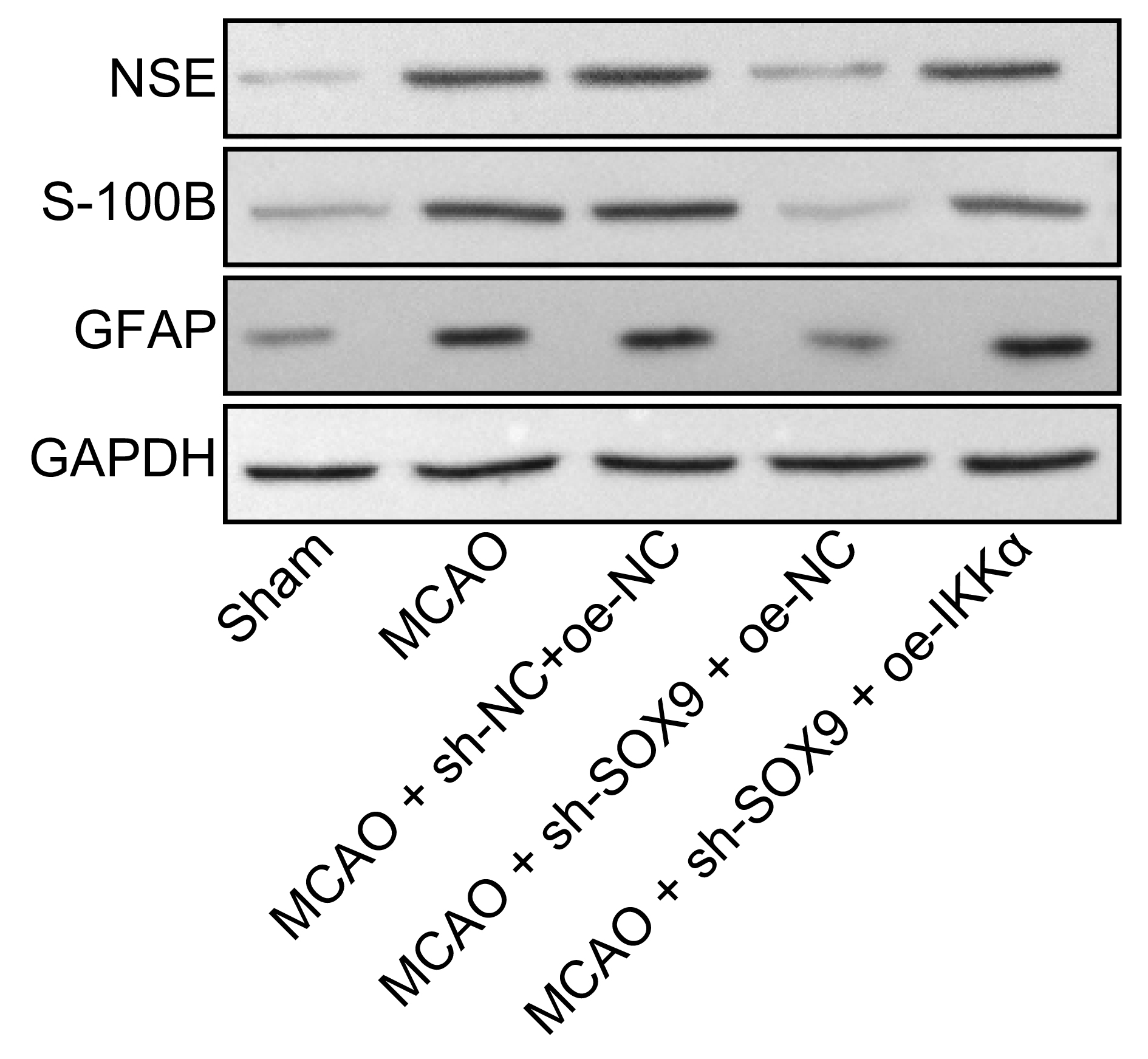

Supplement: Supplementary Figure 2 — SOX9 silencing restrains neuronal damage through upregulating the FOXO3/IKKα in MCAO-operated rats. Western blot analysis of neuronal damage markers (NSE, S-100B, and GFAP) normalized to β-actin in MCAO-operated rats after delivery of lentivirus expressing sh-SOX9 and/or oe-IKKα. [file Image_2.JPEG]
